# Optimization of Mesa Etch for a Quasi-Vertical GaN Schottky Barrier Diode (SBD) by Inductively Coupled Plasma (ICP) and Device Characteristics

**DOI:** 10.3390/nano10040657

**Published:** 2020-04-01

**Authors:** Yue Sun, Xuanwu Kang, Yingkui Zheng, Ke Wei, Pengfei Li, Wenbo Wang, Xinyu Liu, Guoqi Zhang

**Affiliations:** 1 Shenzhen Institute of Wide-Bandgap Semiconductors, Shenzhen 518000, China; y.sun-6@tudelft.nl (Y.S.); wenbo.wang@iwins.org (W.W.); 2 Institute of Microelectronics, Chinese Academy of Sciences, Beijing 100029, China; zhengyingkui@ime.ac.cn (Y.Z.); weike@ime.ac.cn (K.W.); lipengfei@ime.ac.cn (P.L.); xyliu@ime.ac.cn (X.L.); 3 Department of Microelectronics, Delft University of Technology, 2628 CD Delft, The Netherlands

**Keywords:** GaN, inductively coupled plasma (ICP), mesa, sidewall profile, quasi-vertical, Schottky barrier diode (SBD), dry etch

## Abstract

The optimization of mesa etch for a quasi-vertical gallium nitride (GaN) Schottky barrier diode (SBD) by inductively coupled plasma (ICP) etching was comprehensively investigated in this work, including selection of the etching mask, ICP power, radio frequency (RF) power, ratio of mixed gas, flow rate, and chamber pressure, etc. In particular, the microtrench at the bottom corner of the mesa sidewall was eliminated by a combination of ICP dry etching and tetramethylammonium hydroxide (TMAH) wet treatment. Finally, a highly anisotropic profile of the mesa sidewall was realized by using the optimized etch recipe, and a quasi-vertical GaN SBD was demonstrated, achieving a low reverse current density of 10^−8^ A/cm^2^ at −10 V.

## 1. Introduction

**Figure 1:**
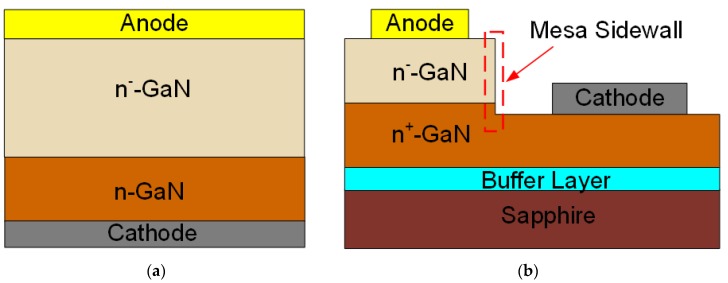
Schematic cross sections of (**a**) vertical gallium nitride (GaN) Schottky barrier diodes (SBDs) and (**b**) quasi-vertical GaN SBDs.

As a wide-bandgap semiconductor material, gallium nitride (GaN) has attracted increasing attention in recent years, attributed to its superior material properties such as wide bandgap, high electron saturation velocity, and high critical electric field [1]. The excellent performance of GaN-based vertical devices has revealed significant potential for high-power and high-frequency applications [2,3]. In addition to the conventional GaN self-standing substrate, epitaxial growth can be carried out on lower-cost and larger-scale foreign substrates (e.g., silicon, sapphire). Therefore, quasi-vertical structures are considered a promising candidate for future GaN-based vertical power devices [4], as shown in Figure 1a,b.

The formation of an active region by the mesa process is the most critical step for quasi-vertical GaN device fabrication. Firstly, the etched sidewall of the mesa was identified as one of the major leakage current paths in quasi-vertical diodes [5]. The leakage current path along the etched sidewall can be reduced by optimization of the etching technique or combination with further surface treatment to reduce plasma damage, leading to improvement of the device breakdown voltage (BV). Secondly, the steep profile of the mesa sidewall allows for reduction of the distance between the anode and cathode electrodes, resulting in a reduction of the series resistance of the Schottky barrier diode (SBD). Therefore, the formation of highly anisotropic profiles of the mesa structure in the etching process is crucial for quasi-vertical GaN devices [6]. Most efforts on the reduction of surface damage and roughness of mesas [7] have been made using inductively coupled plasma (ICP) power, radio frequency (RF) power, the ratio of etching gas, flow rate [8,9,10,11], and mask selection [12]. Moreover, the steepness of the mesa sidewall has also been investigated using ICP dry etching [13]. However, few works have been reported to eliminate microtrench issues for GaN mesa etching [14].

In this paper, we focus on the optimization of the mesa sidewall process to achieve steep, microtrench-free, and low-damage sidewalls, including mask selection, ICP power, RF power, mixed etching gas ratio, flow rate, chamber pressure, etc. Finally, a highly anisotropic profile of the mesa sidewall is realized using the optimized etching recipe, and the device is demonstrated to achieve a low reverse current density under reverse bias. The detailed study is presented below.

## 2. Materials and Methods

**Figure 2:**
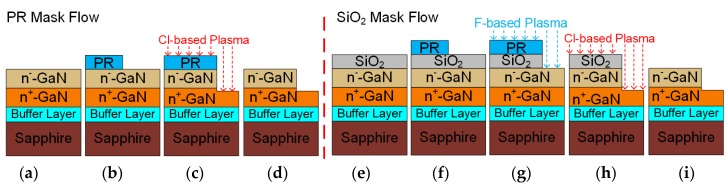
Fabrication steps of mesa etching with photoresist (PR) as a mask. (**a**) Epitaxial structure of the GaN sample; (**b**) PR mask on GaN; (**c**) chlorine-based mesa etching; (**d**) stripping of the PR. Fabrication steps of the SiO_2_-masked GaN mesa etching; (**e**) SiO_2_ hard mask deposition on the GaN sample; (**f**) PR mask on SiO_2_; (**g**) hard mask etching by fluorine-based plasma; (**h**) chlorine-based mesa etching; (**i**) Stripping of the SiO_2_ hard mask.

The samples used in this experiment were all epitaxially grown on 2-inch sapphire (0001) substrates by metalorganic chemical vapor deposition (MOCVD). n-GaN samples with an epitaxial layer thickness of 4.7 μm were used for the etching experiment. Both photoresist (PR) and silicon dioxide (SiO_2_) hard masks were employed for ICP mesa etching in this work. The process flow of mesa etching with the PR mask is illustrated in Figure 2a−d. A thickness of 7 μm positive photoresist (AZ4620) was coated, exposed, and developed for patterning, followed by hard baking (post develop baking) in the oven. Finally, a mesa was formed after chlorine-based ICP dry etching. On the other hand, the process flow of mesa etching with the SiO_2_ hard mask is depicted in Figure 2e−i. A 550 nm SiO_2_ mask layer was firstly deposited by plasma-enhanced chemical vapor deposition (PECVD), followed by 1 μm reversal photoresist (AZ5214E) coating, exposure, and development. The SiO_2_ hard mask was then opened by fluorine-based ICP etching. After the pattern was transferred from the SiO_2_ into the GaN layer and the mesa structure was formed by chlorine-based ICP etching, the SiO_2_ mask was stripped using a buffer of etchant (BOE) solution. In this experiment, the ICP power, RF power, pressure, and ratio of mixed etching gas could be adjusted to obtain highly anisotropic profiles.

Then, the optimized etching recipe was applied for quasi-vertical GaN SBD fabrication. The GaN epi-layer was grown on 2-inch sapphire (0001) and consisted of a 2 μm undoped buffer layer, a 3 μm n^+^-GaN conducting layer (Si doping: 5 × 10^18^ cm^−3^), and a 1 μm n^−^-GaN drift layer (Si doping: 1×10^16^ cm^−3^). After the mesa structure was formed by ICP etching, samples were immersed in tetramethylammonium hydroxide (TMAH) at 45 °C for 30 min. Then, Ti/Al/Ni/Au was deposited and annealed to form ohmic contacts with the n^+^-GaN layer, which acted as the cathode electrode. Finally, a Ni/Au electrode with a radius of 70 μm was deposited to form a Schottky contact with the n^−^-GaN drift layer, which acted as the anode electrode.

## 3. Results and Discussion

Cl_2_ was used as a primary etching gas for the PR-masked etching samples [15]. To investigate the impact of ICP and RF power on the etched sidewall profile, the chamber pressure, flow rate of Cl_2_, and etching time were kept identical. The detailed etching parameters and etching rate results are listed in Table 1.

**Figure 3:**
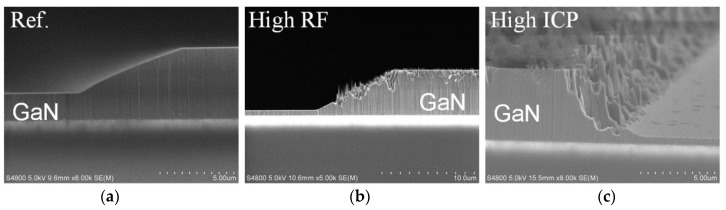
Cross scanning electron microscope (SEM) photomicrographs of GaN mesa sidewalls etched with a PR mask under Cl_2_ plasma conditions with 1.5 Pa pressure and 130 sccm flow rate. (**a**) Reference sample. (**b**) High RF-power etched sample. (**c**) High ICP-power etched sample.

The etching rate of GaN was observed to increase from 120 nm/min to 550 nm/min when the ICP power varied from 360 W to 540 W. This increased etching rate is mainly attributed to the highly reactive chlorine ion (chemical component) density increasing under high ICP power [16]. In addition, the etching rate of GaN was observed to be positively proportional to the RF power as well. The etching rate increased from 120 nm/min at 63 W to 537 nm/min at 210 W. The increased RF power enhances the physical sputtering of GaN etching by heavy radicals; then, the enhanced physical bombardment helps to break the Ga–N bonds, speeding up the chemical etching process [17,18]. As shown in Figure 3a, the reference sample was observed to have a smooth sidewall profile. However, numerous pillars emerged on the sidewall for samples with high RF and those with high ICP power, as shown in Figure 3b,c. Rawal et al. also reported similar observations that mesa edges were damaged by chlorine ions caused by BCl_3_/Cl_2_ discharge during ICP etching [16]. This is due to the photoresist mask erosion caused by enhanced physical bombardment and chemical components; the distorted pattern is then transferred to the GaN, resulting in deteriorated mesa sidewall morphology [9].

**Figure 4:**
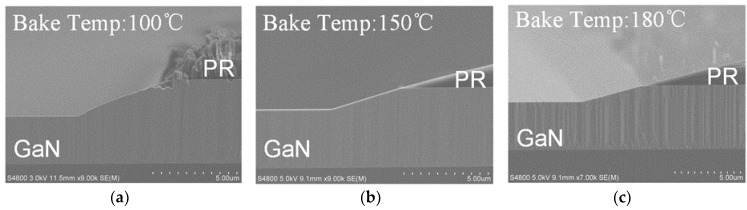
Cross SEM sidewall profiles of photoresist (AZ4620)-masked GaN samples after etching with different hard baking temperatures. (**a**) 100 °C; (**b**) 150 °C; (**c**) 180 °C.

A high-temperature hard baking (post-develop baking) prior to the ICP etching can help to eliminate the PR burning/erosion issues [19]. The baking temperature was varied from 100 °C to 180 °C, followed by ICP etching under ICP power of 360 W, RF power of 63 W, pressure of 1.5 Pa, and Cl_2_ flow rate of 130 sccm. The detailed results are listed in Table 2. As shown in Figure 4a–c, the slope of the mesa sidewall decreased with increasing PR baking temperature, which is due to edge bulge formation at the photoresist pattern as the baking temperature increased, resulting in photoresist sidewall profile transformation and pattern transfer onto the GaN [20,21]. Although baking at a high temperature can help to obtain a smooth mesa sidewall, there are a few drawbacks when the baking temperature applied is above the glass transition temperature (120–130 °C) of the photoresist, such as significant oblique sidewall profile formation, hard striping [22], and volume loss of PR [23].

**Figure 5:**
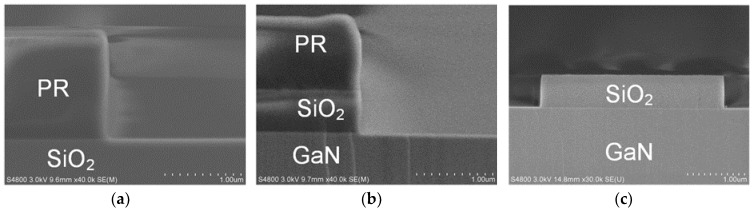
Cross SEM images of the sidewall of (**a**) photoresist (AZ5214E) and of (**b**) SiO_2_ hard mask with PR on top and (**c**) without PR.

Although a smooth mesa sidewall was obtained with the PR mask, a steep sidewall is necessary to reduce the series resistance of a quasi-vertical SBD. A hard mask is more suitable for deep etching of GaN devices, because the etching selectivity of GaN/hard mask is relatively high [12]. To achieve a steep mesa sidewall, we used SiO_2_ as the hard mask in all of the following experiments. Figure 5a shows a cross section of photoresist (AZ5214E) after development of the mesa pattern. Figure 5b,c shows a cross-section of the SiO_2_ hard mask with PR on top and without PR, respectively, after fluorine-based ICP etching of the SiO_2_ hard mask.

**Figure 6:**
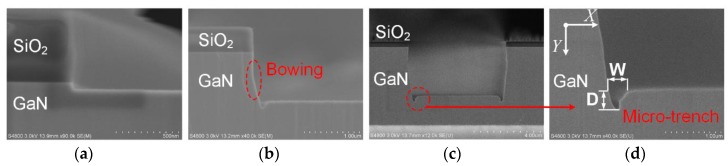
(**a**–**c**) SEM images of sidewall profiles of SiO_2_-masked GaN with different etch times: (**a**) 30 s; (**b**) 7 min; (**c**) 20 min. The marked corner was chosen to show the details of the microtrench. (**d**) SEM image of the microtrench at the bottom corner of the mesa. D and W are the depth and width of the microtrench, respectively.

The impact of etching time on the mesa sidewall profile, etching rates, and etching selectivity was also investigated, while keeping the ICP power, RF power, pressure, and flow rate of Cl_2_ at 360 W, 63 W, 1.5 Pa, and 130 sccm, respectively. The cross sections of sidewalls with varying etching time are shown in Figure 6. With increasing etching time, a microtrench emerged at the bottom corner of the mesa sidewall, as shown in Figure 6b,c. In addition, a slight bowing problem was observed at the mesa sidewall after 7 min of etching. The bowing phenomenon is related with a variety of factors, e.g., non-directional incident ions and isotropic spontaneous chemical etching [24].

**Figure 7:**
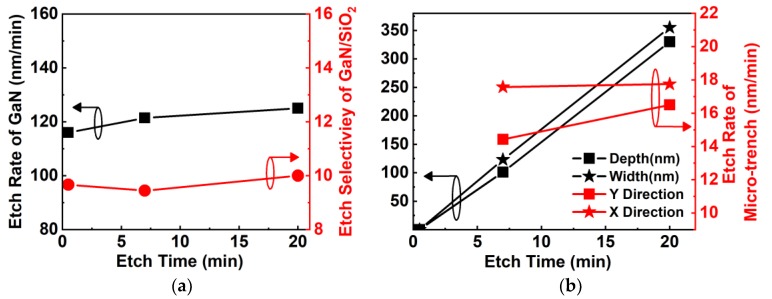
(**a**) Etching rate of GaN and etching selectivity of GaN over SiO_2_ as a function of etching time. (**b**) The effect of etching time on the depth and width of the microtrench, and the etching rates of the microtrench in the X and Y directions.

Figure 7a shows the GaN etching rates and etching selectivity of GaN/SiO_2_ with etching time. Both etching rate and selectivity have a weak dependency on etching time, with values of about 120 and 9.8 nm/min, respectively. As shown in Figure 7b, with increasing etching time, the depth and width of the microtrench increased. The etching rate of the microtrench along the Y direction slightly increased with etching time, and the etching rate along the X direction showed nearly no change. A number of ions were incident along the bowing sidewall and then focused on the bottom corner of the sidewall, leading to an increased etching rate in the Y direction of the microtrench after a long etching time [24,25].

**Figure 8:**
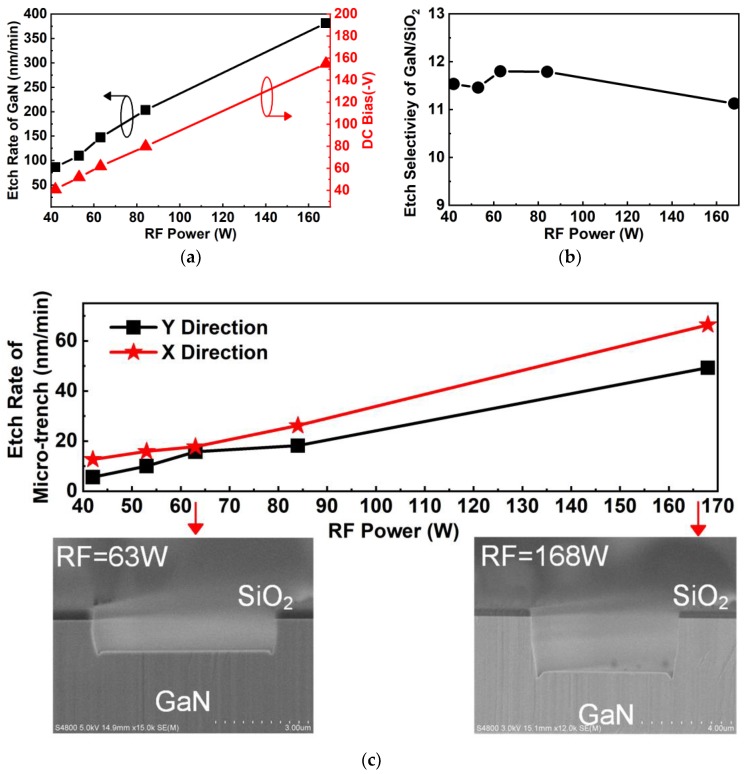
(**a**) Etching rate of GaN and direct current (DC) bias as a function of RF power. (**b**) Etching selectivity of GaN over SiO_2_ as a function of RF power. (**c**) The effect of RF power on etching rates of the microtrench in the X and Y directions. The insets of (**c**) show cross SEM images of samples etched with 63 W and 168 W RF power.

As seen in Figure 8a, the etching rate of GaN is a strong function of RF power and increased from 86 nm/min to 380 nm/min when RF power increased from 42 W to 168 W at 1.5 Pa pressure, 360 W ICP power, and a Cl_2_ flow rate of 130 sccm. With increased RF power from 42 W to 168 W, the direct current (DC) bias increased from −40 V to −155 V. The increased DC bias increases the energy of ions and enhances the physical etching components, which can further increase the chemical etching component [16,26]. Figure 8b shows that the etching selectivity of GaN over SiO_2_ was reduced slightly as RF power increased. As shown in Figure 8c, the etching rate of the microtrench in either the X or Y direction increased with increasing RF power due to the strong physical bombardment [14]. With a low RF power of 63 W, the microtrench could be reduced. However, it deteriorated when the RF power increased to 168 W, as shown in the inset.

**Figure 9:**
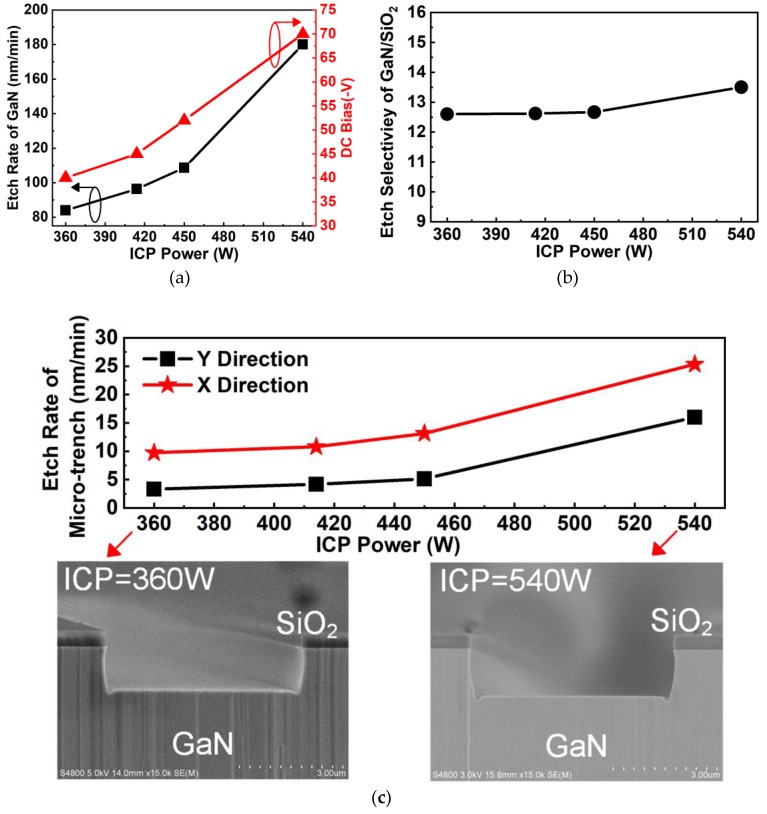
(**a**) Etching rate of GaN and DC bias as a function of ICP power. (**b**) Etching selectivity of GaN over SiO_2_ as a function of ICP power. (**c**) The effect of ICP power on the etching rates of the microtrench in the X and Y directions. The insets of (**c**) show cross SEM images for samples etched with 360 W and 540 W ICP power.

As shown in Figure 9a, the etching rate of GaN increased monotonically with increasing ICP power under etching conditions of 42 W RF power, 130 sccm Cl_2_ flow rate, and 1.5 Pa pressure. The etching rate increased from 84 nm/min to 180 nm/min when the ICP power increased from 360 W to 540 W. The increase in the GaN etching rate is attributed to enhanced chemical etching by increased Cl ion flux density [27]. The observation that the DC bias increases with increasing ICP power is mainly due to the ICP tool operating in capacitive coupling mode (called E mode [28]). When the tool is operated in this mode, ion density is low at low ICP power [29]. Cooke et al. [30], Zhou et al. [27], and Qiu et al. [31] reported similar dependences of DC bias on ICP power. Figure 9b shows that the selectivity increased slightly as ICP power increased. As shown in Figure 9c, the etching rates of the microtrench increased from 3 nm/min to 5 nm/min in the Y direction and from 9 nm/min to 13 nm/min in the X direction when the ICP power increased from 360 W to 450 W. Then, the etching rates rocketed to 16 nm/min in the Y direction and 25 nm/min in the X direction at 540 W ICP power. The reason for this is that the sidewall profile becomes bowed at high ICP power when Cl_2_ is used as the etching gas, then the bowed sidewall profile leads to the accumulation of ions at the corner of the mesa bottom, causing an increase in the microtrench etching rate [32].

**Figure 10:**
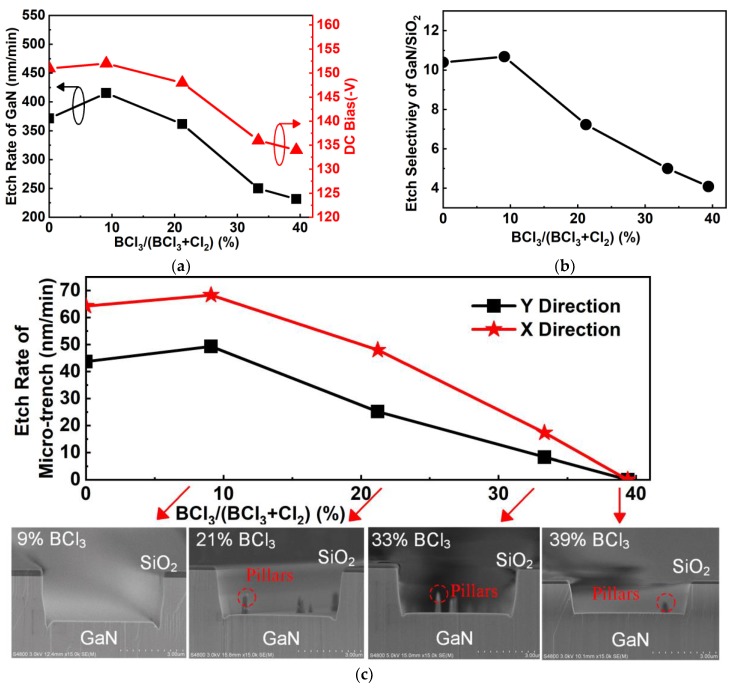
(**a**) Etching rate of GaN and DC bias as a function of BCl_3_ content in the Cl_2_/BCl_3_ gas mixture. (**b**) Etching selectivity of GaN over SiO_2_ as a function of BCl_3_ content in the Cl_2_/BCl_3_ gas mixture. (**c**) The effect of BCl_3_ content in the Cl_2_/BCl_3_ gas mixture on etching rates of the microtrench in the X and Y directions. The insets of (**c**) show cross SEM images for samples etched with different BCl_3_ concentrations.

BCl_3_ as an additive in the etching gas can increase the etching rate of GaN [33]. Thus, in order to improve the etching rate of GaN and investigate the mesa sidewall profile, the percentage of BCl_3_ in the Cl_2_/BCl_3_ gas mixture was varied from 0% to 39% with fixed ICP power of 540 W, RF power of 168 W, total gas flow of 130 sccm, and pressure of 1.5 Pa. Figure 10a shows that the etching rate of GaN increased with increasing BCl_3_ concentration in the Cl_2_/BCl_3_ gas mixture up to 9% BCl_3_, where the maximum etching rate was reached, then decreased from 415 nm/min to 231 nm/min as the BCl_3_ concentration further increased, similar to other reports [34,35]. When the BCl_3_ concentration was below 10%, the Cl radicals and Cl^+^ positive ions showed an increasing tendency in the reaction chamber as the BCl_3_ concentration increased [33], intensifying the ion bombardment and chemical etching process, resulting in the increase of the GaN etching rate. However, the Cl and Cl^+^ ion density decreased with increasing BCl_3_ concentration when the concentration was above 10%, resulting in a reduction of the etching rates of GaN and DC bias [36]. The etching selectivity of GaN over SiO_2_ was significantly reduced from 10.5 to 4 as the BCl_3_ concentration increased (as shown in Figure 10b). This is attributed to the positive BCl_2_^+^ ion density increasing as the BCl_3_ concentration increases, leading to an increase in the SiO_2_ mask etching rate by the formation of BCl_x_O_y_ in the SiO_2_ [37,38]. As shown in Figure 10c, the etching rates of the microtrench slightly increased with the BCl_3_ concentration up to 9%, then decreased further at 33% BCl_3_ and finally vanished at 39% in both the X and Y directions. A significant reduction in the microtrench can be observed in the inset SEM image in Figure 10c as the BCl_3_ concentration increases. The reduction of the microtrench phenomenon is related to the sloped sidewall. With such a sloped sidewall, most ions can be reflected to a position away from the bottom corner of the mesa [39]; thus, less physical bombardment accumulates in the bottom corner of the mesa. The sloped sidewall profile might be attributed to the reduction of etching selectivity of GaN over SiO_2_ in the case of highly anisotropic etching [40]. In addition, pillars were observed in the bottom of the mesa, caused by the spontaneous oxidation of dislocations, inhibiting Cl-based dry etching [41]. In this study, flow rates of 9% BCl_3_ concentration were considered a good trade-off in terms of several aspects, such as etching rate, steepness of the sidewall, pillar phenomenon, and microtrench effect.

**Figure 11:**
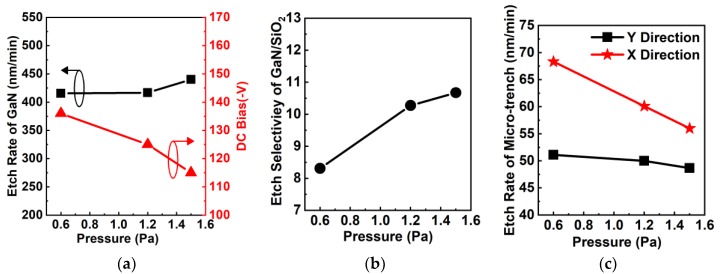
(**a**) Etching rate of GaN and DC bias as a function of pressure. (**b**) Etching selectivity of GaN over SiO_2_ as a function of pressure. (**c**) The effect of pressure on etching rates of the microtrench in the X and Y directions.

Figure 11a shows the impact of chamber pressure on the etching rate of GaN and DC bias. The ICP power, RF power, and flow rates of Cl_2_/BCl_3_ were set at constant values of 540 W, 168 W, and 118 sccm/12 sccm (Cl_2_/9% BCl_3_), respectively. The etching rate of GaN slightly increased from 416 nm/min to 440 nm/min with pressure up to 1.5 Pa, and DC bias was decreased as the pressure increased, reaching a minimum value of ‒115 V at 1.5 Pa. When pressure increases, the mean free path of reactive radicals is reduced, followed by an increase of inter-atom collision ionization [14,27]. Therefore, the etching rate of GaN increased with increasing ion flux and the DC bias decreased as the RF power was kept at a constant value [30]. As shown in Figure 11b, the etching selectivity of GaN/SiO_2_ increased monotonically as the chamber pressure increased, similar to findings by Wang et al. [42]. Figure 11c shows that etching rates of the microtrench along both the X and Y directions decreased with increased pressure. The etching rate in the X direction has higher dependency on chamber pressure than that in the Y direction, which is attributed to the irregular distribution of incoming ion angles [39]. Therefore, high chamber pressure is preferred to obtain high etching selectivity.

**Figure 12:**
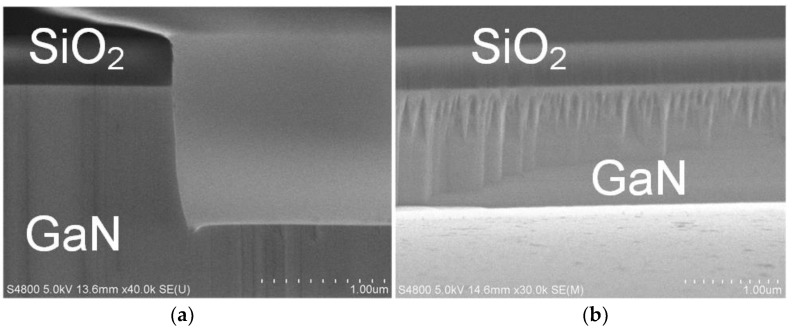

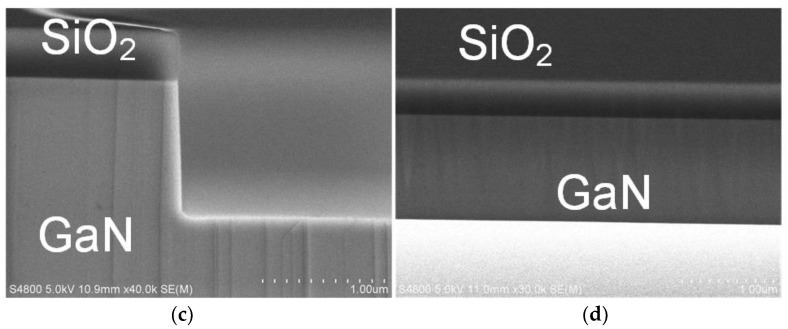
SEM micrographs of SiO_2_-masked GaN etched with the optimum ICP etching recipe before tetramethylammonium hydroxide (TMAH) wet treatment. (**a**) Cross-sectional view of the sidewall; (**b**) sidewall surface. After TMAH wet treatment: (**c**) cross-sectional view of the sidewall; (**d**) sidewall surface.

The GaN mesa structure was finalized with optimized conditions of 360 W ICP power, 42 W RF power, 118 sccm/12 sccm flow rate of Cl_2_/BCl_3_ mixture gas, and 1.5 Pa pressure, approaching a near-90° steep sidewall with a tiny microtrench and a slightly rough surface, as shown in Figure 12a,b. The etching rate of GaN was near 120 nm/min and the selectivity of GaN over SiO_2_ was 10. The etching depth was near 1.2 μm by adjusting etching time. Figure 12c,d shows cross SEM images of the sidewall and surface after dipping in 15% TMAH solution at 45℃ for 30 min. The sidewall was near 90° steep and the surface was smoother after TMAH wet treatment. This is due to the sidewall being etched preferentially in anisotropic wet etching solution [43,44,45].

**Figure 13:**
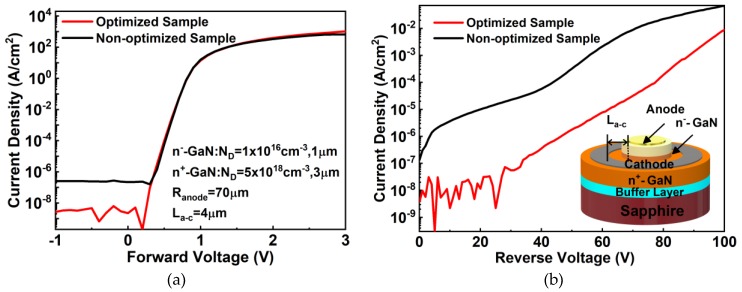
(**a**) Forward and (**b**) reverse *I–V* characteristics of the optimized and non-optimized quasi-vertical GaN SBDs in this study. The inset of (**b**) shows a 3D schematic of the SBD. R_anode_ and L_a-c_ are the anode radius and the distance between the anode edge and cathode, respectively.

Finally, quasi-vertical GaN-on-sapphire SBDs with a 1 μm n^−^-GaN drift layer and a 3 μm n^+^-GaN conducting layer were fabricated with a 1.2 μm deep mesa. The forward and reverse characteristics are shown in Figure 13a,b, respectively. Both of the samples achieved a high forward current density of 1000 A/cm^2^ at 3 V, while the optimized sample had a reverse leakage current density of 10^−8^ A/cm^2^ at −10 V, which is 2 orders of magnitude lower than that of the non-optimized one. The ideality factor of nearly 1 indicates less of a trapping effect, both in bulk and interface. Moreover, the state-of-the-art low reverse leakage current indicates that the damage to the mesa sidewall is low [46,47,48,49]. The leakage current in this work is much lower than that in our previous work with a low-damage etching technique, in which a device was fabricated based on AlGaN/GaN heterostructures [50]. Therefore, we suggest that the smooth and low-damage mesa sidewall etching and wet treatment probably contribute to this extremely low leakage current [5].

## 4. Conclusions

In summary, the influence of ICP etching conditions on GaN mesa sidewall profiles was studied and quasi-vertical GaN SBDs were fabricated using the optimized recipe. For the PR-masked GaN samples, high ICP power and RF power are the causes of deteriorated mesa sidewall morphology. Although high-temperature (>140 °C) hard baking prior to etching can produce a smooth sidewall, the drawbacks are significant oblique sidewall profile formation and hard striping. For the SiO_2_-masked GaN samples, the etching rate of GaN is dependent on the ICP power, RF power, and ratio of BCl_3_/Cl_2_ gas flow, but has relatively less dependence on the chamber pressure. The etching selectivity of GaN over SiO_2_ decreases with increasing BCl_3_ concentration in Cl_2_/BCl_3_ mixture gas in the range of 0–40%. The etching selectivity of GaN/SiO_2_ can be increased by adjusting the chamber pressure. Moreover, the microtrench problem at the bottom corner of the mesa can be reduced or eliminated by reducing the ICP power or RF power or by adding BCl_3_ into the Cl_2_ plasma. After ICP etching, the use of a TMAH wet treatment for samples can obtain a near-90° steep mesa sidewall that is microtrench free and has a smooth sidewall surface.

Therefore, the optimized ICP etching recipe is as follows: 360 W ICP power, 42 W RF power, 118 sccm/12 sccm Cl_2_/BCl_3_ flow rates, 1.5 Pa chamber pressure, and subsequent treatment with 15% concentration TMAH at 45 °C for 30 min. Finally, quasi-vertical GaN-on-sapphire SBDs with 1.2 μm mesa depth were fabricated using the optimized etching recipe, and the device characteristics were demonstrated to achieve a low reverse leakage current density of 10^−8^ A/cm^2^ at −10 V, suggesting that the damage to the mesa sidewall is low.

## Figures and Tables

**Table 1:** Result of PR-masked GaN samples under different etching conditions.

Sample	ICP Power (W)	RF Power (W)	Pressure (Pa)	Cl_2_ (sccm)	GaN Etching Rate (nm/min)	θ_GaN_
Reference	360	63	1.5	130	120	25.0°
High RF	360	210	1.5	130	537	-
High ICP	540	63	1.5	130	550	-

θ_GaN_ is the etched GaN sidewall angle. RF power is the radio frequency power. ICP power is the inductively coupled plasma power.

**Table 2:** Etched results with different hard baking temperatures.

Sample	ICP Power (W)	RF Power (W)	Pressure (Pa)	Cl_2_ (sccm)	GaN Etching Rate (nm/min)	θ_GaN_	T (℃)
1	360	63	1.5	130	135	23.0°	100
2	360	63	1.5	130	123	17.0°	150
3	360	63	1.5	130	117	15.0°	180

θ_GaN_ is the etched GaN sidewall angle. T is the photoresist hard baking temperature.
